# Primary Non-Adherence and Associated Factors in Patients with Atrial Fibrillation in Germany: A Retrospective Cohort Study

**DOI:** 10.3390/jcdd13100499

**Published:** 2026-10-03

**Authors:** Karel Kostev, Beate Leppert, Maximilian Peters, Henning Sievert, Marcel Konrad, Mark Luedde

**Affiliations:** 1 University Hospital, University of Marburg, 35043 Marburg, Germany; 2 Epidemiology, IQVIA, 60597 Frankfurt am Main, Germany; 3 Real-World Data Analytics, IQVIA, 80331 Munich, Germany; 4 Real-World Solutions, IQVIA, 60597 Frankfurt am Main, Germany; 5 Health & Social, FOM University of Applied Sciences for Economics and Management, 60486 Frankfurt am Main, Germany; 6 Department of Cardiology and Angiology, University Hospital of Giessen, 35392 Giessen, Germany; 7 Department of Internal Medicine and Cardiology and Angiology, Christian-Albrechts-University of Kiel, 24118 Kiel, Germany

**Keywords:** atrial fibrillation, primary non-adherence, oral anticoagulants, vitamin K antagonists, direct oral anticoagulants, medication adherence, prescriptions

## Abstract

Background: Primary non-adherence (PNA)—failure to dispense a newly prescribed medication—is an early barrier to effective treatment. This study aimed to estimate PNA to oral anticoagulants in patients with atrial fibrillation (AF) in Germany and to identify associated factors. Methods: In this retrospective cohort study, first prescriptions of vitamin K antagonists (VKAs), direct thrombin inhibitors, or direct factor Xa inhibitors issued to AF patients between 2016 and 2025 in the IQVIA Disease Analyzer database were combined with pharmacy dispensing records in the Longitudinal Prescription (LRx) database. First prescriptions preceded by a dispensing of the same class within 365 days were excluded. PNA was defined as failure to dispense within 30 days; associated factors were assessed using logistic regression with generalized estimating equations. Results: The study included 57,290 first prescriptions in 53,815 patients (mean age 75.5 years; 49.7% women). PNA occurred in 10.6% (VKAs 14.4%, direct thrombin inhibitors 11.7%, direct factor Xa inhibitors 10.1%). Direct factor Xa inhibitors (odds ratio [OR] 0.67; 95% confidence interval [CI] 0.62–0.72), prior myocardial infarction (OR 0.79), and dementia (OR 0.85) were associated with lower odds of PNA, whereas ischemic heart disease (OR 1.18) and hypertension (OR 1.11) were associated with higher odds. Non-dispensing of the first prescription was strongly associated with non-dispensing of subsequent prescriptions. Conclusions: Approximately one in ten first oral anticoagulant prescriptions in AF was not dispensed within 30 days, most frequently for VKAs.

## 1. Introduction

Atrial fibrillation (AF) is the most common sustained cardiac arrhythmia, and its prevalence rises steeply with age. Global burden analyses have documented a continued increase in the number of people living with AF and atrial flutter over recent decades, a trend that is particularly pronounced in ageing European populations [1,2]. AF is a leading risk factor for ischemic stroke and systemic embolism, and its management therefore centers on long-term stroke prevention [3].

Current European guidelines recommend oral anticoagulation for stroke prevention in most patients with AF and give preference to direct oral anticoagulants (DOACs)—comprising direct thrombin inhibitors and direct factor Xa inhibitors—over vitamin K antagonists (VKAs), reflecting their more favorable safety profile, fixed dosing, and lack of routine coagulation monitoring [3]. The stroke-preventive benefit of anticoagulation, however, depends not only on appropriate prescribing but also on patients actually obtaining and continuing to use the prescribed medication.

Primary non-adherence (PNA)—the failure to dispense an initially prescribed medication—has been described as an early and often overlooked barrier to effective treatment that precedes any subsequent adherence or persistence problem, and that may prevent the initiation of evidence-based therapy altogether [4]. Population-based studies in general primary care have reported PNA rates of approximately 14–17% for new prescriptions overall, with substantial variation by drug class, patient age, and prescriber characteristics [5,6], while a review of PNA to cardiovascular pharmacotherapy reported an overall rate exceeding 10% [7], and a systematic review of initial non-adherence to antihypertensive medications reported estimates ranging from 5% to 34% [8].

For oral anticoagulants in AF, most real-world evidence to date has addressed ongoing (secondary) adherence and persistence—typically measured through the proportion of days covered or discontinuation over months to years—rather than the earlier step of whether the first prescription is dispensed at all [9,10,11]. The few available studies of PNA to anticoagulants in AF have been conducted predominantly in North American settings and have reported markedly higher non-dispensing than for cardiovascular drugs in general [12,13], but comparable data from European statutory health-care systems, and analyses comparing anticoagulant classes, are scarce. This study therefore aimed to estimate the rate of PNA to oral anticoagulants in AF patients in Germany—overall, by drug class, and by individual agent—among first prescriptions of each anticoagulant class, and to identify patient-level factors associated with PNA, using a large primary care database combined with pharmacy dispensing records; in addition, non-dispensing of subsequent prescriptions and associated factors were examined.

## 2. Materials and Methods

### 2.1. Data Sources

This retrospective cohort study was based on a combination of two IQVIA databases, the Longitudinal Prescription (LRx) database and the Disease Analyzer (DA) database. The LRx database comprises approximately 80% of prescriptions reimbursed by statutory health insurance funds in Germany. Data are available at the patient level and include information on age and sex. All patient data are fully anonymized by the data provider in accordance with applicable data protection regulations. Each prescription record contains detailed product information (e.g., brand, active substance, package size, and formulation) as well as the dispensing date. The LRx database does not contain information on diagnoses or laboratory values [14].

The DA database contains anonymized data on diagnoses, prescriptions, and basic sociodemographic characteristics collected from general and specialized outpatient practices in Germany. Practices are selected based on predefined criteria, including physician age, specialty, federal state, and community size, and the database has been shown to be representative of outpatient practices in Germany [15]. Depending on physician specialty, database coverage ranges from approximately 3% to 7%.

### 2.2. Study Population and Outcomes

This retrospective cohort study included patients aged ≥ 18 years with a documented diagnosis of atrial fibrillation (International Classification of Diseases, 10th Revision [ICD-10]: I48) in general and internal medicine practices contributing to the DA database. The unit of analysis was the first prescription of an oral anticoagulant class—vitamin K antagonists (VKAs; phenprocoumon, warfarin), direct thrombin inhibitors (dabigatran), or direct factor Xa inhibitors (apixaban, edoxaban, rivaroxaban)—issued between 2016 and 2025, defined as a prescription without any earlier prescription of the same class in the DA database. Because anticoagulation may have been initiated by other physicians, for example at hospital discharge or in cardiology practices, and then continued in general practice, a first prescription in the DA database does not necessarily represent treatment initiation. First prescriptions were therefore only included if no dispensing of the same anticoagulant class was recorded in the LRx database, which captures dispensing irrespective of the prescribing physician, in the 365 days before the prescription date. Prior use of a different anticoagulant class was permitted, as a switch represents the initiation of a new therapy; patients could therefore contribute one first prescription for each class they started. The AF diagnosis had to be documented within the five years before the first anticoagulant prescription. In addition, all anticoagulant prescriptions following each patient’s first anticoagulant prescription during the study period (subsequent prescriptions) were analyzed separately in all AF patients with at least one anticoagulant prescription.

The study outcome was PNA, defined as failure to dispense a drug of the same anticoagulant class within 30 days of the prescription date (day 0 to day 30). This window is consistent with previous studies of PNA to oral anticoagulants in AF [12,13], and windows of 14, 60, and 90 days were examined in sensitivity analyses. Patients from the DA database were combined with records from the LRx database using a validated co-therapy-based approach. Using this method we analyzed patients across both databases based on identical prescription patterns (i.e., the same drugs prescribed on the same dates), without the use of personal identifiers, ensuring full anonymization and compliance with data protection requirements. For each prescription, dispensing of a drug of the same anticoagulant class within the respective window was ascertained in the LRx database; in a further sensitivity analysis, dispensing was ascertained at the level of the individual agent. Non-dispensing of subsequent prescriptions was ascertained analogously using the 30-day window. To ensure sufficient observation time, only prescriptions with pharmacy activity extending more than 30 days (or more than 60 and 90 days in the respective sensitivity analyses) beyond the prescription date were retained.

### 2.3. Statistical Analyses

Characteristics at the date of each first prescription were described using means and standard deviations (SDs) for age and absolute numbers and percentages for categorical variables. These included age groups (≤50, 51–60, 61–70, 71–80, and >80 years), sex, anticoagulant class and individual agent, whether the prescription was the patient’s first anticoagulant prescription or followed a switch from another class, chronic comorbidities documented within the five years before the prescription date (heart failure, ischemic heart disease, myocardial infarction, hypertension, dyslipidemia, type 2 diabetes mellitus, depression, obesity, cancer, chronic obstructive pulmonary disease [COPD], history of stroke or transient ischemic attack [TIA], and dementia), and cardiovascular co-medication (diuretics, beta-blockers, calcium channel blockers, renin–angiotensin–aldosterone system [RAAS] inhibitors, and lipid-lowering agents) prescribed in the 12 months before the prescription date. In addition, the CHA_2_DS_2_-VASc score [16] and a modified HAS-BLED score [17] were approximated from documented diagnoses and prescriptions (Table S1). The modified HAS-BLED score did not include labile international normalized ratio (INR), as laboratory values were not available, and hypertension was defined by a documented diagnosis rather than by uncontrolled blood pressure.

The proportion of PNA was evaluated descriptively, overall and stratified by anticoagulant class, individual agent, age group, sex, comorbidities, co-medication, and score categories. Because patients could contribute more than one first prescription, factors associated with PNA were assessed using multivariable logistic regression with generalized estimating equations (GEE; independent working correlation, robust standard errors, clustering by patient). The main model included anticoagulant class (direct thrombin inhibitors and direct factor Xa inhibitors, with VKAs as reference) as the primary variable of interest and was adjusted for age group (reference: 71–80 years), sex, comorbidities, co-medication, and first prescription after a switch. To examine whether the association with age was linear, age was additionally modelled as a continuous variable (per 10 years). Further models replaced anticoagulant class by individual agents (VKAs as reference), compared twice-daily (apixaban, dabigatran) with once-daily (edoxaban, rivaroxaban) DOACs, replaced the individual score components by CHA_2_DS_2_-VASc (0–2, 3–4, ≥5) and modified HAS-BLED (0–1, 2, ≥3) categories, and extended the main model by vascular disease, renal disease, liver disease, history of bleeding, alcohol abuse, antiplatelet agents, and nonsteroidal anti-inflammatory drugs (NSAIDs). Sensitivity analyses varied the dispensing window (14, 60, and 90 days), ascertained dispensing at the level of the individual agent, restricted the analysis to one first prescription per patient, and repeated the analysis without the exclusion of first prescriptions preceded by a dispensing of the same class. Results are reported as odds ratios (OR) with 95% confidence intervals (CI); the main model is displayed as a forest plot.

Time from prescription to the first dispensing of the same class was described up to 365 days. Non-dispensing of subsequent prescriptions was described by prescription order and by time since the first prescription and analyzed using a GEE model including anticoagulant class, prescription order, time since the first prescription, non-dispensing of the patient’s first prescription, and the covariates listed above. Switches between anticoagulant classes were described, including whether the last prescription of the previous class had been dispensed.

We acknowledge that other factors of potential relevance to PNA—such as smoking, socioeconomic status, cognitive function beyond a documented dementia diagnosis, distance between physician practice and pharmacy, and out-of-pocket costs—were not available in the database and therefore could not be included. Consequently, the selected variables should be interpreted as a set of clinically relevant and data-available covariates rather than a comprehensive representation of all factors related to PNA, and residual confounding cannot be excluded. Given the number of covariates examined and the very large sample size, a conservative significance threshold of *p* < 0.01 was applied; in the forest plot, statistically significant associations (*p* < 0.01) are shown in blue and non-significant associations (*p* ≥ 0.01) in grey. Analyses were conducted using SAS version 9.4 (SAS Institute, Cary, NC, USA).

## 3. Results

### 3.1. Basic Characteristics of the Study Sample

The study included 57,290 first anticoagulant prescriptions in 53,815 AF patients (Table 1). Of these, 51,707 (90.3%) were the patient’s first anticoagulant prescription and 5583 (9.7%) were first prescriptions of a new class after a switch; 3397 patients (6.3%) contributed first prescriptions of two or three classes. The mean age at the first prescription was 75.5 years (SD 10.9), and 49.7% of the first prescriptions were issued to women. Direct factor Xa inhibitors accounted for 86.8% of first prescriptions (apixaban 48.0%, edoxaban 20.1%, rivaroxaban 18.7%), followed by VKAs (9.8%) and direct thrombin inhibitors (3.4%). Of 87,803 AF patients with at least one anticoagulant prescription in the DA database, the first recorded prescription had been preceded by a dispensing of the same class within 365 days in 36,095 (41.1%), indicating continuation of a therapy initiated elsewhere; these prescriptions were not counted as first prescriptions.

Hypertension (60.5%), dyslipidemia (30.1%), and type 2 diabetes mellitus (26.8%) were the most prevalent comorbidities, followed by ischemic heart disease (23.5%) and heart failure (22.1%); dementia was documented for 6.4% of first prescriptions. Beta-blockers (73.4%) and RAAS inhibitors (67.4%) were the most frequently co-prescribed cardiovascular medications. The mean CHA_2_DS_2_-VASc score was 3.5 (SD 1.7), and the mean modified HAS-BLED score was 2.0 (SD 1.0) (Table 1).

### 3.2. Primary Non-Adherence Rates

PNA occurred in 6055 of 57,290 first prescriptions (10.6%) (Table 2). By anticoagulant class, crude PNA was highest for VKAs (14.4%) and lower for direct thrombin inhibitors (11.7%) and direct factor Xa inhibitors (10.1%). Among individual agents, PNA was lowest for apixaban (9.6%) and edoxaban (9.6%) and higher for dabigatran (11.7%) and rivaroxaban (11.9%). First prescriptions after a switch from another class were less often not dispensed than patients’ first anticoagulant prescriptions (8.8% vs. 10.8%). For comparison, non-dispensing was 4.7% for the 36,095 first recorded prescriptions preceded by a dispensing of the same class, and 8.3% for the first recorded prescription of all 87,803 patients without this exclusion. Non-dispensing of subsequent prescriptions was 7.1% (Table 2).

Across subgroups, PNA ranged from approximately 9% to 14% (Table 2). PNA was highest in patients aged ≤ 50 years (13.2%) and similar across the age groups from 61 years onward (10.0–10.8%). It was similar in women (10.7%) and men (10.5%) and somewhat lower for prescriptions to patients with prior myocardial infarction (8.6%), dementia (8.8%), and a history of stroke or TIA (9.5%). PNA was slightly lower at higher CHA_2_DS_2_-VASc scores (11.2% for 0–2 vs. 10.0% for ≥5) and higher modified HAS-BLED scores (11.6% for 0–1 vs. 9.2% for ≥3).

Most first prescriptions were dispensed promptly: 65.1% on the day of prescription, 83.2% within three days, and 89.4% within 30 days (Table S3). Only 0.6% were dispensed between day 31 and day 60. Of the 6055 first prescriptions not dispensed within 30 days, 2847 (47.0%) were followed by a dispensing of the same class between day 31 and day 365, whereas for 3208 (5.6% of all first prescriptions) no dispensing of the same class was recorded within one year. With a 14-, 60-, and 90-day window, PNA was 11.6%, 10.0%, and 9.2%, respectively, and ascertainment of dispensing at the level of the individual agent yielded a PNA of 10.7% (Table S2).

### 3.3. Factors Associated with Primary Non-Adherence

**Figure 1:**
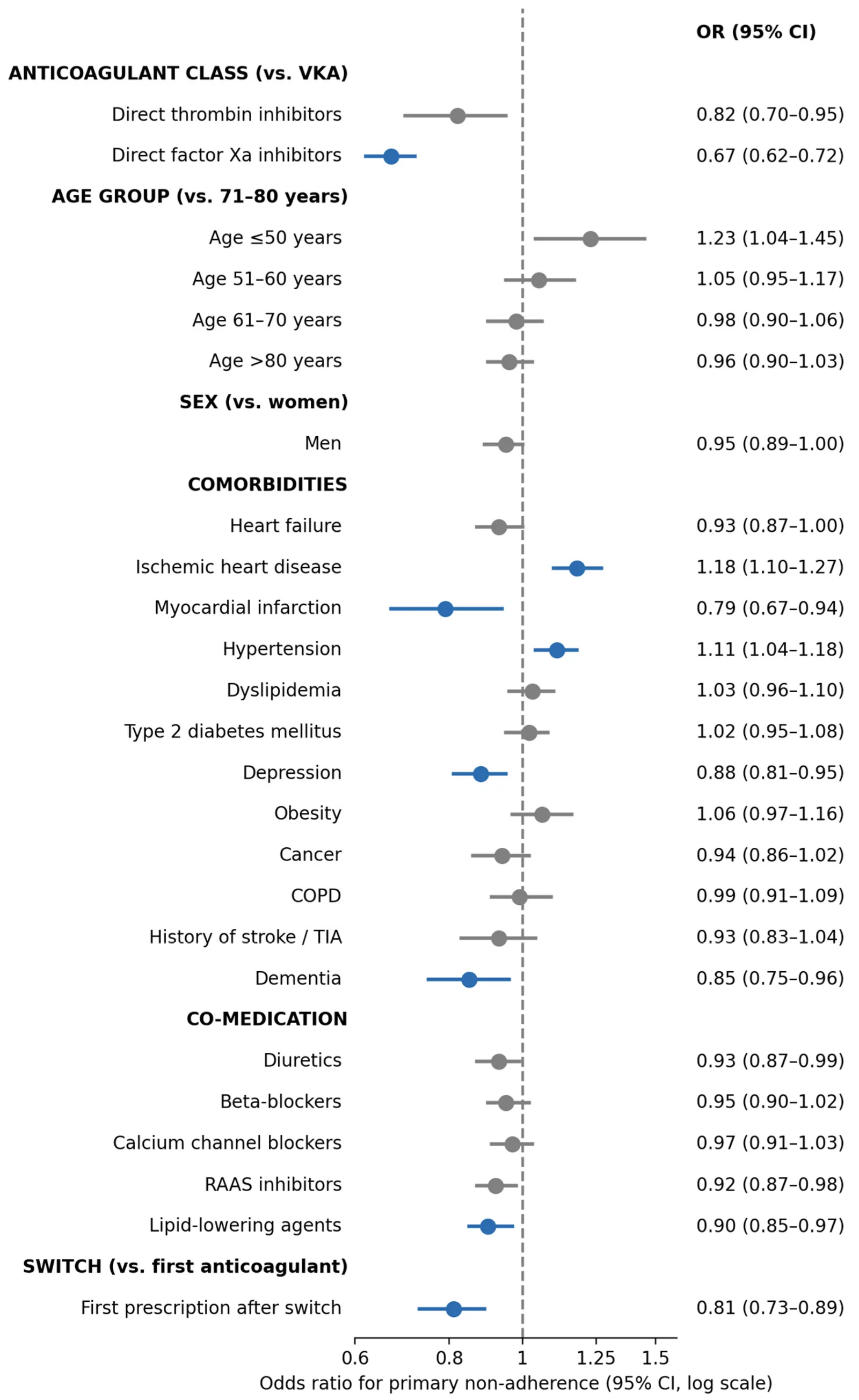
Forest plot of the multivariable logistic regression model (generalized estimating equations, clustering by patient) for primary non-adherence at the first prescription of an anticoagulant class in AF patients (57,290 first prescriptions in 53,815 patients). Symbols show odds ratios and horizontal lines the 95% confidence intervals on a logarithmic scale; the dashed line marks an odds ratio of 1. Statistically significant associations (*p* < 0.01) are shown in blue and non-significant associations (*p* ≥ 0.01) in grey. Reference categories (odds ratio = 1): vitamin K antagonists, age 71–80 years, women, and patients’ first anticoagulant prescription. COPD: chronic obstructive pulmonary disease; TIA: transient ischemic attack; RAAS: renin–angiotensin–aldosterone system.

In the multivariable GEE model (57,290 first prescriptions; Figure 1), direct factor Xa inhibitors were associated with substantially lower odds of PNA than VKAs (OR 0.67; 95% CI 0.62–0.72); the association for direct thrombin inhibitors was in the same direction (OR 0.82; 95% CI 0.70–0.95; *p* = 0.011). Compared with patients aged 71–80 years, the odds of PNA were numerically higher in patients aged ≤ 50 years (OR 1.23; 95% CI 1.04–1.45; *p* = 0.015), without reaching the prespecified significance level, and the other age groups, including patients aged > 80 years (OR 0.96; 95% CI 0.90–1.03), did not differ; modelled continuously, the odds of PNA were 4% lower per 10-year increase in age (OR 0.96; 95% CI 0.93–0.99). Ischemic heart disease (OR 1.18; 95% CI 1.10–1.27) and hypertension (OR 1.11; 95% CI 1.04–1.18) were associated with higher odds of PNA, whereas prior myocardial infarction (OR 0.79; 95% CI 0.67–0.94), depression (OR 0.88; 95% CI 0.81–0.95), dementia (OR 0.85; 95% CI 0.75–0.96), and prior use of lipid-lowering agents (OR 0.90; 95% CI 0.85–0.97) were associated with lower odds. First prescriptions after a switch had lower odds of PNA than patients’ first anticoagulant prescriptions (OR 0.81; 95% CI 0.73–0.89). The association with male sex was weak and not significant (OR 0.95; 95% CI 0.89–1.00).

Compared with VKAs, the odds of PNA were lowest for apixaban (OR 0.64; 95% CI 0.59–0.69) and edoxaban (OR 0.64; 95% CI 0.58–0.70) and higher for rivaroxaban (OR 0.78; 95% CI 0.71–0.86) and dabigatran (OR 0.82; 95% CI 0.70–0.95) (Table S4). Among DOACs, twice-daily agents were associated with slightly lower odds of PNA than once-daily agents (OR 0.92; 95% CI 0.87–0.97). When the individual components were replaced by risk scores, a modified HAS-BLED score ≥ 3 was associated with lower odds of PNA (OR 0.79; 95% CI 0.72–0.86; reference 0–1), whereas the association with a CHA_2_DS_2_-VASc score ≥ 5 was weak and not significant (OR 1.12; 95% CI 1.02–1.23; reference 0–2; *p* = 0.020). In the extended model, prior use of antiplatelet agents (OR 0.81; 95% CI 0.75–0.88) and NSAIDs (OR 0.80; 95% CI 0.74–0.86) was associated with lower odds of PNA, whereas vascular disease, renal disease, liver disease, history of bleeding, and alcohol abuse were not significantly associated (Table S4). The associations with anticoagulant class were consistent across all sensitivity analyses, with ORs between 0.59 and 0.70 for direct factor Xa inhibitors and between 0.75 and 0.83 for direct thrombin inhibitors (Table S2).

### 3.4. Non-Dispensing of Subsequent Prescriptions and Treatment Switching

Among 77,477 patients with at least one subsequent prescription (median 9 subsequent prescriptions per patient, interquartile range [IQR] 4–16), non-dispensing was 7.7% for the second prescription and 7.0% for the sixth and later prescriptions; it was 7.6% in the first three months after the first prescription and between 6.9% and 7.5% thereafter (Table S5). In the GEE model, longer time since the first prescription was associated with lower odds of non-dispensing (OR 0.77; 95% CI 0.69–0.84 for >24 months vs. 0–3 months), whereas prescription order was not associated with non-dispensing after accounting for time. Non-dispensing of the first prescription showed the strongest association with later non-dispensing (OR 12.47; 95% CI 11.84–13.13): 72.5% of patients whose first prescription had not been dispensed also failed to dispense at least one subsequent prescription, compared with 15.3% of patients whose first prescription had been dispensed. Age ≤ 50 years (OR 1.44; 95% CI 1.22–1.70), age 51–60 years (OR 1.15; 95% CI 1.05–1.26), and prior use of calcium channel blockers (OR 1.08; 95% CI 1.02–1.15) were associated with higher odds of non-dispensing, whereas age > 80 years (OR 0.85; 95% CI 0.80–0.90), dementia (OR 0.68; 95% CI 0.60–0.76), direct factor Xa inhibitors (OR 0.81; 95% CI 0.75–0.88), and direct thrombin inhibitors (OR 0.83; 95% CI 0.71–0.95) were associated with lower odds.

Overall, 6456 of 87,803 patients (7.4%) switched to another anticoagulant class during the study period, after a median of 671 days (IQR 222–1425). Most switches were from VKAs to direct factor Xa inhibitors (65.5%), followed by switches from direct factor Xa inhibitors to VKAs (13.2%) and from direct thrombin inhibitors to direct factor Xa inhibitors (12.0%) (Table S6). In 11.5% of switches, the last prescription of the previous class had not been dispensed. The first anticoagulant prescription had more often not been dispensed in patients who later switched than in patients who did not switch (12.3% vs. 7.9%).

## 4. Discussion

In this large real-world study of AF patients in Germany, approximately one in ten first prescriptions of an oral anticoagulant class (10.6%) was not dispensed within 30 days. PNA was highest for VKAs (14.4%) and lowest for apixaban and edoxaban (9.6% each). Most first prescriptions were dispensed on the day of prescription, and almost half of the prescriptions not dispensed within 30 days were dispensed later, indicating delayed rather than definitively forgone treatment in a considerable proportion of patients. A methodologically relevant finding is that 41% of the first anticoagulant prescriptions recorded in general and internal medicine practices had been preceded by a dispensing of the same class, most likely reflecting therapy initiated in hospitals or cardiology practices. Including these continued therapies would have underestimated PNA (8.3% instead of 10.6%). Studies of PNA based on prescriber data should therefore verify that the prescription indeed represents the initiation of treatment.

The observed PNA is lower than the 14–17% reported for new prescriptions in general primary care [5,6] and substantially lower than the 21–28% reported for oral anticoagulants in North American AF cohorts [12,13], and it is within the range reported for cardiovascular pharmacotherapy [7,8]. This difference coincides with several characteristics of the German health-care system. Statutory health insurance covers almost 90% of the population and reimburses all oral anticoagulants without prior authorization; co-payments amount to 10% of the price, with a minimum of EUR 5 and a maximum of EUR 10 per package, and are capped at 2% of the annual household income (1% for the chronically ill). In the United States, by contrast, out-of-pocket costs, insurance type, and social factors have been identified as important correlates of primary non-dispensing of oral anticoagulants [12,13]. The pattern across agents also differs: in the study by Hernandez et al., non-dispensing was numerically lowest for warfarin [12], an inexpensive generic drug, whereas VKAs had the highest PNA in our study. This contrast is compatible with the hypothesis that costs are relevant for the initial dispensing of DOACs in the United States, whereas in Germany the practical requirements of VKA therapy may be more relevant; however, this hypothesis cannot be tested with the present data. Continuity of care with the general practitioner, a dense pharmacy network, and differences in data sources and definitions may also be relevant for the lower rates observed in Germany.

The lower PNA for DOACs than for VKAs was consistent across all sensitivity analyses and parallels the better ongoing adherence and persistence generally reported for DOACs than for VKAs in real-world AF populations, including in Germany [9,11], although cross-national European data indicate that adherence and persistence vary between individual agents and settings [18]. The greater practical burden associated with initiating VKA therapy, including the need for routine coagulation monitoring and dose titration, may be one factor related to the higher non-dispensing observed for this class. Among DOACs, PNA was lowest for apixaban and edoxaban, which is consistent with the numerically lowest non-dispensing for apixaban among DOACs in the United States [12]. Contrary to the expectation that once-daily dosing would be associated with lower PNA, twice-daily agents were not associated with higher PNA; the slightly lower odds for twice-daily agents mainly reflect the low PNA observed for apixaban. Dosing frequency was therefore not strongly associated with initial dispensing, although it may be more relevant for ongoing adherence.

Patients aged ≤ 50 years had numerically higher PNA, although this association did not reach the prespecified significance level; a higher non-dispensing in younger patients has also been reported in general primary care [6] and for oral anticoagulants [12]. After subdivision of patients aged > 70 years, however, the oldest patients (>80 years) did not differ from those aged 71–80 years, and the association with age was weak overall (OR 0.96 per 10 years); thus, there was no indication of lower initial dispensing among the oldest and potentially frail patients. The lower PNA in patients with dementia may be related to medication management by relatives or nursing services. Prior myocardial infarction was associated with lower PNA, which is compatible with the more intensive cardiovascular follow-up of these patients, whereas ischemic heart disease and hypertension were associated with slightly higher PNA; patients with these conditions frequently already use several cardiovascular drugs, which may be related to a lower willingness to start an additional long-term medication, although this interpretation cannot be tested with the present data. Higher CHA_2_DS_2_-VASc scores were not associated with lower PNA, which is in line with the higher non-dispensing observed in patients with a CHA_2_DS_2_-VASc score ≥ 3 in the United States [12]. The lower PNA in patients with a high modified HAS-BLED score and in users of antiplatelet agents or NSAIDs may be related to more frequent medical contacts and established routines of medication use. In patients with competing risks, such as those with cancer, the balance between thromboembolic and bleeding risk is particularly complex [19]. As higher CHA_2_DS_2_-VASc scores were not associated with lower PNA and a high bleeding risk score was associated with lower rather than higher PNA, the present data do not indicate a strong association between the estimated stroke or bleeding risk and a lower likelihood of initial dispensing. As an observational analysis, these findings describe associations rather than causal relationships.

Non-dispensing of subsequent prescriptions (7.1%) was lower than PNA and lower at longer treatment duration, whereas the number of previous prescriptions was not associated with non-dispensing once time was taken into account. This complements the secondary-adherence literature, in which discontinuation of oral anticoagulation tends to cluster in the early months after initiation [10,11]. Later non-dispensing was most strongly associated with non-dispensing of the first prescription, as almost three quarters of these patients also failed to dispense at least one later prescription. PNA was therefore associated with a persistently higher likelihood of non-dispensing during follow-up. In line with the decrease in non-dispensing from 28.4% at 30 days to 17% at 180 days reported by Hernandez et al. [12], almost half of the first prescriptions not dispensed within 30 days were dispensed later, indicating delayed initiation of stroke prevention. Switches between anticoagulant classes occurred in 7% of patients, mostly from VKAs to direct factor Xa inhibitors, and approximately one in nine switches followed a non-dispensed prescription, indicating that switches were in some cases preceded by non-dispensing; the reasons for switching, however, are not documented in the databases.

From a clinical perspective, PNA to oral anticoagulants in Germany is lower than reported from other settings, and system-wide interventions do not appear warranted. Nevertheless, approximately one in ten patients does not start the prescribed stroke prevention in time, and 5.6% of first prescriptions were not followed by any dispensing of the same class within one year. Pragmatic measures could therefore focus on the first weeks after the initial prescription: explaining the rationale for anticoagulation at the time of prescribing, particularly when VKAs are initiated; verifying at the next contact that anticoagulation has actually been started; and paying particular attention to patients who did not dispense their first prescription, as non-dispensing of the first prescription was strongly associated with further non-dispensing. The increasing use of electronic prescriptions in Germany may facilitate such follow-up in the future.

To our knowledge, this is among the first studies to examine PNA to oral anticoagulants in AF within a European statutory health-care setting, focusing on first prescriptions of each anticoagulant class, verifying treatment initiation with pharmacy dispensing records, and comparing anticoagulant classes and individual agents in the same large population. Most previous AF-specific adherence research has focused on ongoing adherence and persistence to DOACs using indirect measures such as the proportion of days covered [9,10,11,18], leaving the earlier and arguably more fundamental step of initial dispensing comparatively underexamined, particularly outside North America [12,13].

This study has several strengths, including the large, real-world sample size, the use of validated, linked pharmacy dispensing data [14,15], the verification of treatment initiation, and the robustness of the main findings across several sensitivity analyses. Several limitations should nonetheless be acknowledged. As an observational study, the findings reflect associations rather than causal relationships, and residual confounding cannot be excluded. The CHA_2_DS_2_-VASc and HAS-BLED scores could only be approximated from documented diagnoses and prescriptions; the modified HAS-BLED score did not include labile INR, and hypertension was defined by diagnosis rather than by blood pressure values. Socioeconomic status, out-of-pocket costs, the distance between practice and pharmacy, and cognitive function beyond a documented dementia diagnosis were not available. The LRx database covers approximately 80% of prescriptions reimbursed by statutory health insurance, and privately insured patients are not represented. Prescriptions were captured from general and internal medicine practices; anticoagulation initiated in hospitals or cardiology practices is not directly captured, and prescriptions continuing such therapy were excluded when a dispensing of the same class preceded the first prescription in the DA database. The findings therefore refer to the initiation of treatment in primary care, and primary adherence after hospital discharge or in specialist care may differ. The 365-day lookback period may not have identified all patients with earlier use of the same class, and medication obtained outside public pharmacies, for example in hospital, could not be captured. Finally, the data provide no information on the reasons for non-dispensing or switching, nor on whether dispensed medications were taken as prescribed.

## 5. Conclusions

In this large real-world study of AF patients in Germany, approximately one in ten first prescriptions of an oral anticoagulant class was not dispensed within 30 days, and about half of these were dispensed later. PNA was highest for VKAs and lowest for apixaban and edoxaban, whereas age and dosing frequency showed only weak associations. Non-dispensing of the first prescription was strongly associated with later non-dispensing. Although PNA was lower than reported from North American settings, verifying treatment initiation after the first anticoagulant prescription, particularly in patients starting VKAs, could be considered to identify delayed or forgone stroke prevention at an early stage. Future research should examine whether PNA is associated with clinical outcomes in AF.

## Figures and Tables

**Table 1:** Characteristics of AF patients at the date of the first prescription of an anticoagulant class.

Variable	First Prescriptions (N = 57,290)
Number of first prescriptions	57,290
Number of patients	53,815
Type of first prescription, n (%)	
First anticoagulant prescription of the patient	51,707 (90.3)
First prescription after switch from another class	5583 (9.7)
Age, years (mean, SD)	75.5 (10.9)
Age group, n (%)	
≤50 years	1417 (2.5)
51–60 years	4406 (7.7)
61–70 years	10,531 (18.4)
71–80 years	19,217 (33.5)
>80 years	21,719 (37.9)
Sex, n (%)	
Women	28,468 (49.7)
Men	28,822 (50.3)
Anticoagulant class, n (%)	
Vitamin K antagonists	5623 (9.8)
Direct thrombin inhibitors	1967 (3.4)
Direct factor Xa inhibitors	49,700 (86.8)
Individual agent, n (%)	
Phenprocoumon/warfarin	5623 (9.8)
Dabigatran	1967 (3.4)
Apixaban	27,504 (48.0)
Edoxaban	11,497 (20.1)
Rivaroxaban	10,699 (18.7)
Comorbidities, n (%)	
Hypertension	34,646 (60.5)
Dyslipidemia	17,230 (30.1)
Type 2 diabetes mellitus	15,356 (26.8)
Ischemic heart disease	13,471 (23.5)
Heart failure	12,659 (22.1)
Depression	7809 (13.6)
Cancer	7266 (12.7)
COPD	6209 (10.8)
Obesity	5935 (10.4)
History of stroke/TIA	4131 (7.2)
Dementia	3684 (6.4)
Myocardial infarction	1860 (3.2)
Further score components, n (%)	
Vascular disease	9321 (16.3)
History of bleeding	4240 (7.4)
Alcohol abuse	1021 (1.8)
Renal disease	728 (1.3)
Liver disease	583 (1.0)
Co-medication (prior 12 months), n (%)	
Beta-blockers	42,054 (73.4)
RAAS inhibitors	38,600 (67.4)
Diuretics	26,313 (45.9)
Lipid-lowering agents	24,113 (42.1)
Calcium channel blockers	17,092 (29.8)
Antiplatelet agents	12,033 (21.0)
NSAIDs	11,212 (19.6)
CHA_2_DS_2_-VASc score, mean (SD)	3.5 (1.7)
0–2	16,044 (28.0)
3–4	26,119 (45.6)
≥5	15,127 (26.4)
Modified HAS-BLED score, mean (SD)	2.0 (1.0)
0–1	16,638 (29.0)
2	22,949 (40.1)
≥3	17,703 (30.9)

Characteristics were determined at the date of each first prescription; percentages refer to first prescriptions. First prescriptions preceded by a dispensing of the same class within 365 days were excluded. SD: standard deviation; COPD: chronic obstructive pulmonary disease; TIA: transient ischemic attack; RAAS: renin–angiotensin–aldosterone system; NSAIDs: nonsteroidal anti-inflammatory drugs. The modified HAS-BLED score does not include labile INR. Definitions of all variables are given in Table S1.

**Table 2:** Primary non-adherence (PNA) at the first prescription of an anticoagulant class in AF patients, overall and by subgroup; non-dispensing of subsequent prescriptions shown separately.

Subgroup	N	Not Dispensed, n (%)
Primary non-adherence—first prescriptions (n = 57,290)		
Total	57,290	6055 (10.6)
Anticoagulant class		
Vitamin K antagonists	5623	812 (14.4)
Direct thrombin inhibitors	1967	231 (11.7)
Direct factor Xa inhibitors	49,700	5012 (10.1)
Individual agent		
Phenprocoumon/warfarin	5623	812 (14.4)
Dabigatran	1967	231 (11.7)
Apixaban	27,504	2638 (9.6)
Edoxaban	11,497	1106 (9.6)
Rivaroxaban	10,699	1268 (11.9)
Type of first prescription		
First anticoagulant prescription of the patient	51,707	5564 (10.8)
First prescription after switch	5583	491 (8.8)
Age group		
≤50 years	1417	187 (13.2)
51–60 years	4406	501 (11.4)
61–70 years	10,531	1118 (10.6)
71–80 years	19,217	2070 (10.8)
>80 years	21,719	2179 (10.0)
Sex		
Women	28,468	3032 (10.7)
Men	28,822	3023 (10.5)
Comorbidities		
Heart failure	12,659	1229 (9.7)
Ischemic heart disease	13,471	1483 (11.0)
Myocardial infarction	1860	160 (8.6)
Hypertension	34,646	3729 (10.8)
Dyslipidemia	17,230	1814 (10.5)
Type 2 diabetes mellitus	15,356	1599 (10.4)
Depression	7809	737 (9.4)
Obesity	5935	651 (11.0)
Cancer	7266	709 (9.8)
COPD	6209	637 (10.3)
History of stroke/TIA	4131	393 (9.5)
Dementia	3684	323 (8.8)
Cardiovascular co-medication		
Diuretics	26,313	2610 (9.9)
Beta-blockers	42,054	4352 (10.3)
Calcium channel blockers	17,092	1729 (10.1)
RAAS inhibitors	38,600	3935 (10.2)
Lipid-lowering agents	24,113	2398 (9.9)
CHA_2_DS_2_-VASc score		
0–2	16,044	1796 (11.2)
3–4	26,119	2739 (10.5)
≥5	15,127	1520 (10.0)
Modified HAS-BLED score		
0–1	16,638	1938 (11.6)
2	22,949	2485 (10.8)
≥3	17,703	1632 (9.2)
Non-dispensing of subsequent prescriptions (n = 865,637)		
Total	865,637	61,891 (7.1)
Anticoagulant class		
Vitamin K antagonists	53,715	5083 (9.5)
Direct thrombin inhibitors	37,815	2614 (6.9)
Direct factor Xa inhibitors	774,107	54,194 (7.0)

PNA: no dispensing of the same anticoagulant class within 30 days of the first prescription; N = number of first prescriptions, n = number not dispensed. For subsequent prescriptions (all prescriptions following each patient’s first anticoagulant prescription among 87,803 AF patients with at least one anticoagulant prescription), N = number of subsequent prescriptions and n = number not dispensed within 30 days. % = n/N. Subgroups other than anticoagulant class, agent, and prescription type are not mutually exclusive. COPD: chronic obstructive pulmonary disease; TIA: transient ischemic attack; RAAS: renin–angiotensin–aldosterone system.

## Data Availability

The data used in this study are subject to strict privacy and confidentiality requirements and therefore cannot be made publicly available. Legal restrictions prohibit the sharing of the raw data with third parties.

## Supplementary Materials

The following supporting information can be downloaded at: https://www.mdpi.com/article/10.3390/jcdd13100499/s1, Table S1: Definitions of variables and risk scores; Table S2: Sensitivity analyses; Table S3: Time from prescription to dispensing; Table S4: Individual agents, dosing frequency, risk scores, and additional covariates; Table S5: Non-dispensing of subsequent prescriptions; Table S6: Switching between anticoagulant classes.

## Abbreviations

The following abbreviations are used in this manuscript:

AF	Atrial fibrillation
CI	Confidence interval
COPD	Chronic obstructive pulmonary disease
DA	Disease Analyzer
DOAC	Direct oral anticoagulant
GEE	Generalized estimating equations
ICD-10	International Classification of Diseases, 10th Revision
INR	International normalized ratio
IQR	Interquartile range
LRx	Longitudinal Prescription database
NSAID	Nonsteroidal anti-inflammatory drug
OR	Odds ratio
PNA	Primary non-adherence
RAAS	Renin–angiotensin–aldosterone system
SD	Standard deviation
TIA	Transient ischemic attack
VKA	Vitamin K antagonist

## References

[1] Lippi G., Sanchis-Gomar F., Cervellin G. (2021). Global epidemiology of atrial fibrillation: An increasing epidemic and public health challenge. Int. J. Stroke.

[2] Cheng S., He J., Han Y., Han S., Li P., Liao H., Guo J. (2024). Global burden of atrial fibrillation/atrial flutter and its attributable risk factors from 1990 to 2021. Europace.

[3] Van Gelder I.C., Rienstra M., Bunting K.V., Casado-Arroyo R., Caso V., Crijns H.J.G.M., De Potter T.J.R., Dwight J., Guasti L., Hanke T. (2024). 2024 ESC Guidelines for the management of atrial fibrillation developed in collaboration with the European Association for Cardio-Thoracic Surgery (EACTS). Eur. Heart J..

[4] Chapman S.C.E., Chan A.H.Y. (2025). Medication nonadherence—Definition, measurement, prevalence, and causes: Reflecting on the past 20 years and looking forwards. Front. Pharmacol..

[5] Zeitouny S., Cheng L., Wong S.T., Tadrous M., McGrail K., Law M.R. (2023). Prevalence and predictors of primary nonadherence to medications prescribed in primary care. CMAJ.

[6] Aznar-Lou I., Fernández A., Gil-Girbau M., Fajó-Pascual M., Moreno-Peral P., Peñarrubia-María M.T., Serrano-Blanco A., Sánchez-Niubó A., March-Pujol M.A., Jové A.M. (2017). Initial medication non-adherence: Prevalence and predictive factors in a cohort of 1.6 million primary care patients. Br. J. Clin. Pharmacol..

[7] Schulz M., Laufs U. (2024). Not obtaining a medication the first time it is prescribed: Primary non-adherence to cardiovascular pharmacotherapy. Clin. Res. Cardiol..

[8] Cooke C.E., Xing S., Gale S.E., Peters S. (2022). Initial non-adherence to antihypertensive medications in the United States: A systematic literature review. J. Hum. Hypertens..

[9] Ozaki A.F., Choi A.S., Le Q.T., Ko D.T., Han J.K., Park S.S., Jackevicius C.A. (2020). Real-world adherence and persistence to direct oral anticoagulants in patients with atrial fibrillation: A systematic review and meta-analysis. Circ. Cardiovasc. Qual. Outcomes.

[10] Fuchs V.A., Spinewine A., Zerah L., Henrard S. (2025). Non-adherence and non-persistence of direct oral anticoagulants among patients with non-valvular atrial fibrillation: A French retrospective cohort study. Sci. Rep..

[11] Patti G., Wang R., Marston X.L., Yeh Y.C., Zimmermann L., Ye X., Gao X., Brüggenjürgen B. (2023). Anticoagulant treatment adherence and persistence in German patients with atrial fibrillation. Cardiol. Ther..

[12] Hernandez I., Divino V., Xie L., Hood D.W., DeKoven M., Kariuki W., Bell G., Russ C., Cheng D., Cato M. (2023). A real-world evaluation of primary medication nonadherence in patients with nonvalvular atrial fibrillation prescribed oral anticoagulants in the United States. Am. J. Cardiovasc. Drugs.

[13] Glowacki N., Guzman I., Mills J.R., Parks A., Ateya M., Dai F., Dorscheid H., Khatib R. (2025). Impact of social determinants of health on primary adherence of oral anticoagulants among patients with newly diagnosed atrial fibrillation. Cardiol. Ther..

[14] Kostev K., Wang Y., Singh R., Kalder M., Loosen S.H., Roderburg C., Konrad M., Jacob L. (2024). German longitudinal prescription database (LRx): Description of characteristics, use in pharmacoepidemiological research, and limitations. Int. J. Clin. Pharmacol. Ther..

[15] Rathmann W., Bongaerts B., Carius H.J., Kruppert S., Kostev K. (2018). Basic characteristics and representativeness of the German Disease Analyzer database. Int. J. Clin. Pharmacol. Ther..

[16] Lip G.Y.H., Nieuwlaat R., Pisters R., Lane D.A., Crijns H.J.G.M. (2010). Refining clinical risk stratification for predicting stroke and thromboembolism in atrial fibrillation using a novel risk factor-based approach: The Euro Heart Survey on atrial fibrillation. Chest.

[17] Pisters R., Lane D.A., Nieuwlaat R., de Vos C.B., Crijns H.J.G.M., Lip G.Y.H. (2010). A novel user-friendly score (HAS-BLED) to assess 1-year risk of major bleeding in patients with atrial fibrillation: The Euro Heart Survey. Chest.

[18] Sabaté M., Vidal X., Ballarin E., Rottenkolber M., Schmiedl S., Grave B., Huerta C., Martin-Merino E., Montero D., Leon-Muñoz L.M. (2021). Adherence to direct oral anticoagulants in patients with non-valvular atrial fibrillation: A cross-national comparison in six European countries (2008–2015). Front. Pharmacol..

[19] Mauriello A., Correra A., Quagliariello V., Iovine M., Di Micco P., Imbalzano E., Giallauria F., Giordano A., Russo V., D’Andrea A. (2025). Atrial fibrillation and cancer: Pathophysiological mechanism and clinical implications. J. Clin. Med..

