# Supplementary material for: Primary Non-Adherence and Associated Factors in Patients with Atrial Fibrillation in Germany: A Retrospective Cohort Study
Source: J Cardiovasc Dev Dis. 2026 Oct 3;13(10):499. doi: 10.3390/jcdd13100499 (PMC13704982; doi:10.3390/jcdd13100499)
Supplement: Supplementary file 1 [file jcdd-13-00499-s001.zip › jcdd-4571903-supplementary.pdf]

Supplementary Materials

Primary Non-Adherence and Associated Factors in Patients with Atrial Fibrillation in Germany: A Retrospective Cohort Study

Karel Kostev, Beate Leppert, Maximilian Peters, Henning Sievert, Marcel Konrad and Mark Luedde

Table S1. Definitions of variables and risk scores.

Variable	Definition (ICD-10 codes or ATC codes)
**Study population and exposure**	
Atrial fibrillation	I48
Vitamin K antagonists	ATC B01A (phenprocoumon, warfarin)
Direct thrombin inhibitors	ATC B01E (dabigatran)
Direct factor Xa inhibitors	ATC B01F (apixaban, edoxaban, rivaroxaban)
**Comorbidities (main model)**	
Heart failure	I50
Ischemic heart disease	I25
Myocardial infarction	I21, I22, I23
Hypertension	I10
Dyslipidemia	E78
Type 2 diabetes mellitus	E10, E11, E14
Depression	F32, F33
Obesity	E66
Cancer	C00–C97
COPD	J44
History of stroke/TIA	I63, G45
Dementia	F00, F01, F02, F03, G30
**Components of the risk scores**	
Congestive heart failure	I50, I11.0, I13.0, I13.2
Hypertension	I10, I11, I12, I13, I15
Diabetes mellitus	E10, E11, E12, E13, E14
Stroke, TIA, or thromboembolism	I63, I64, G45, I69.3, I69.4, I74
Vascular disease	I21, I22, I23, I25.2, I70, I71, I73.9
Renal disease	N18.4, N18.5, Z49, Z94.0, Z99.2
Liver disease	K70, K72, K73, K74, K76.6, K76.7
History of bleeding	I60, I61, I62, S06.4, S06.5, S06.6, K92.0, K92.1, K92.2, K62.5, K25–K28 (.0, .2, .4, .6), R04, R31, R58, D62, H35.6, H43.1
Alcohol abuse	F10, K70
**Co-medication (prior 12 months)**	
Diuretics	ATC C03
Beta-blockers	ATC C07
Calcium channel blockers	ATC C08
RAAS inhibitors	ATC C09
Lipid-lowering agents	ATC C10

Antiplatelet agents	ATC B01C
NSAIDs	ATC M01A
**Risk scores**	
CHA_2DS_2-VASc	Congestive heart failure 1; hypertension 1; age ≥75 years 2; diabetes mellitus 1; stroke, TIA, or thromboembolism 2; vascular disease 1; age 65–74 years 1; female sex 1 (maximum 9)
Modified HAS-BLED	Hypertension 1; renal disease 1; liver disease 1; stroke 1; history of bleeding 1; age >65 years 1; antiplatelet agents or NSAIDs 1; alcohol abuse 1 (labile INR not available; maximum 8)

Diagnoses were considered if documented as confirmed diagnoses within the five years before the date of the first prescription; co-medication was considered if prescribed within the 12 months before this date. ATC codes refer to the anatomical classification used in the IQVIA databases. ICD-10: International Classification of Diseases, 10th Revision; ATC: Anatomical Therapeutic Chemical; COPD: chronic obstructive pulmonary disease; TIA: transient ischemic attack; RAAS: renin-angiotensin-aldosterone system; NSAIDs: nonsteroidal anti-inflammatory drugs; INR: international normalized ratio.

Table S2. Sensitivity analyses: primary non-adherence (PNA) and adjusted odds ratios for anticoagulant classes (reference: vitamin K antagonists).

Analysis	N	PNA, n (%)	Direct thrombin inhibitors, OR (95% CI)	Direct factor Xa inhibitors, OR (95% CI)
Main analysis (30-day window; GEE)	57,290	6055 (10.6)	0.82 (0.70–0.95)	0.67 (0.62–0.72)
14-day window	57,290	6645 (11.6)	0.81 (0.70–0.94)	0.66 (0.61–0.71)
60-day window	56,285	5630 (10.0)	0.80 (0.68–0.93)	0.64 (0.59–0.69)
90-day window	55,368	5093 (9.2)	0.75 (0.63–0.88)	0.59 (0.54–0.64)
Dispensing ascertained at the level of the individual agent	57,290	6117 (10.7)	0.82 (0.70–0.95)	0.68 (0.63–0.73)
Risk scores instead of individual components	57,290	6055 (10.6)	0.82 (0.70–0.95)	0.67 (0.62–0.72)
Extended covariate set	57,290	6055 (10.6)	0.83 (0.71–0.96)	0.69 (0.63–0.74)
One first prescription per patient (logistic regression)	51,708	5564 (10.8)	0.78 (0.65–0.92)	0.65 (0.60–0.71)
All first prescriptions per class without exclusion of prior same-class dispensing (GEE)	94,392	7785 (8.2)	0.78 (0.68–0.89)	0.70 (0.66–0.75)
First recorded prescription per patient without exclusion of prior same-class dispensing (logistic regression)	87,803	7244 (8.3)	0.75 (0.65–0.87)	0.69 (0.64–0.74)

All models except the risk score model (see Table S4) were adjusted for age group, sex, comorbidities, and co-medication; models including more than one prescription per patient additionally included first prescription after a switch and used generalized estimating equations (GEE) with clustering by patient. For the 60- and 90-day windows, only prescriptions with more than 60 and 90 days of observation were included. The risk score model replaced the individual score components by CHA_2DS_2-VASc and modified HAS-BLED categories; the extended model additionally included vascular disease, renal disease, liver disease, history of bleeding, alcohol abuse, antiplatelet agents, and NSAIDs. OR: odds ratio; CI: confidence interval.

Table S3. Time from the first prescription to the first dispensing of the same anticoagulant class (N = 57,290).

Time to dispensing	n (%)	Cumulative %

Day of prescription	37,297 (65.1)	65.1
1–3 days	10,388 (18.1)	83.2
4–7 days	2103 (3.7)	86.9
8–14 days	857 (1.5)	88.4
15–30 days	590 (1.0)	89.4
31–60 days	368 (0.6)	90.1
61–90 days	500 (0.9)	90.9
91–365 days	1979 (3.5)	94.4
No dispensing within 365 days	3208 (5.6)	100.0
**First prescriptions not dispensed within 30 days (n = 6055)**		
Dispensed between day 31 and day 365	2847 (47.0)	
No dispensing within 365 days	3208 (53.0)	

Dispensing of a drug of the same anticoagulant class was ascertained in the LRx database. The observation time after the prescription date exceeded 30 days for all first prescriptions but could be shorter than 365 days.

Table S4. Associations of individual agents, dosing frequency, age, risk scores, and additional covariates with primary non-adherence (57,290 first prescriptions).

Variable	OR (95% CI)	p-Value
**Individual agents (reference: phenprocoumon/warfarin)**		
Apixaban	0.64 (0.59–0.69)	<0.001
Edoxaban	0.64 (0.58–0.70)	<0.001
Rivaroxaban	0.78 (0.71–0.86)	<0.001
Dabigatran	0.82 (0.70–0.95)	0.0104
**Dosing frequency among DOACs (reference: once daily)**		
Twice daily (apixaban, dabigatran)	0.92 (0.87–0.97)	0.004
**Age as continuous variable**		
Per 10 years	0.96 (0.93–0.99)	0.003
**Risk score model**		
CHA_2DS_2-VASc 3–4 (reference: 0–2)	1.03 (0.96–1.11)	0.396
CHA_2DS_2-VASc ≥5 (reference: 0–2)	1.12 (1.02–1.23)	0.020
Modified HAS-BLED 2 (reference: 0–1)	0.93 (0.87–1.00)	0.061
Modified HAS-BLED ≥3 (reference: 0–1)	0.79 (0.72–0.86)	<0.001
**Extended model: additional covariates**		
Vascular disease	0.92 (0.84–1.01)	0.067
Renal disease	0.78 (0.59–1.02)	0.074
Liver disease	0.93 (0.69–1.27)	0.670
History of bleeding	0.90 (0.80–1.01)	0.063
Alcohol abuse	0.82 (0.65–1.03)	0.093
Antiplatelet agents	0.81 (0.75–0.88)	<0.001
NSAIDs	0.80 (0.74–0.86)	<0.001

Logistic regression with generalized estimating equations (clustering by patient). All models were adjusted as the main model (age group, sex, comorbidities, co-medication, and first prescription after a switch), with the following exceptions: the model for age as a continuous variable replaced the age groups; the risk score model included anticoagulant class, the score categories,

dyslipidemia, depression, obesity, cancer, COPD, dementia, co-medication, and first prescription after a switch, as age, sex, and the remaining comorbidities are components of the scores. DOACs: direct oral anticoagulants; OR: odds ratio; CI: confidence interval.

Table S5. Non-dispensing of subsequent prescriptions within 30 days (865,637 subsequent prescriptions in 77,477 patients).

Subgroup	N	Not dispensed, n (%)
**Prescription order**		
2nd prescription	77,477	5946 (7.7)
3rd prescription	70,170	5286 (7.5)
4th–5th prescription	123,486	9039 (7.3)
6th–10th prescription	233,344	16,415 (7.0)
>10th prescription	361,160	25,205 (7.0)
**Time since the first prescription**		
0–3 months	54,262	4109 (7.6)
>3–6 months	64,544	4827 (7.5)
>6–12 months	115,993	8397 (7.2)
>12–24 months	184,615	12,823 (6.9)
>24 months	446,223	31,735 (7.1)
**Individual agent**		
Phenprocoumon/warfarin	53,715	5083 (9.5)
Dabigatran	37,815	2614 (6.9)
Apixaban	405,922	28,048 (6.9)
Edoxaban	184,345	12,562 (6.8)
Rivaroxaban	183,840	13,584 (7.4)
**First prescription dispensed within 30 days (patient level)**		
Yes: patients with ≥1 non-dispensed subsequent prescription	70,907	10,864 (15.3)
No: patients with ≥1 non-dispensed subsequent prescription	6570	4764 (72.5)

Variable (GEE model)	OR (95% CI)	p-Value
**Anticoagulant class (reference: vitamin K antagonists)**		
Direct thrombin inhibitors	0.83 (0.71–0.95)	0.0096
Direct factor Xa inhibitors	0.81 (0.75–0.88)	<0.001
**Prescription order (reference: 2nd prescription)**		
3rd prescription	1.03 (0.99–1.07)	0.167
4th–5th prescription	1.04 (0.98–1.09)	0.186
6th–10th prescription	1.02 (0.95–1.10)	0.549
>10th prescription	0.97 (0.88–1.07)	0.502
**Time since the first prescription (reference: 0–3 months)**		
>3–6 months	0.94 (0.90–0.98)	0.006

>6–12 months	0.87 (0.82–0.92)	<0.001
>12–24 months	0.80 (0.74–0.87)	<0.001
>24 months	0.77 (0.69–0.84)	<0.001
**First prescription**		
Not dispensed within 30 days	12.47 (11.84–13.13)	<0.001
**Age group (reference: 71–80 years)**		
≤50 years	1.44 (1.22–1.70)	<0.001
51–60 years	1.15 (1.05–1.26)	0.004
61–70 years	0.99 (0.92–1.06)	0.688
>80 years	0.85 (0.80–0.90)	<0.001
**Sex (reference: women)**		
Men	1.04 (0.99–1.09)	0.133
**Comorbidities**		
Heart failure	0.98 (0.91–1.04)	0.478
Ischemic heart disease	1.01 (0.95–1.07)	0.835
Myocardial infarction	0.95 (0.80–1.12)	0.530
Hypertension	0.97 (0.92–1.02)	0.278
Dyslipidemia	0.97 (0.92–1.03)	0.374
Type 2 diabetes mellitus	0.98 (0.93–1.04)	0.488
Depression	1.02 (0.95–1.10)	0.610
Obesity	1.01 (0.93–1.09)	0.860
Cancer	1.05 (0.97–1.13)	0.265
COPD	0.98 (0.90–1.06)	0.641
History of stroke/TIA	1.00 (0.90–1.10)	0.957
Dementia	0.68 (0.60–0.76)	<0.001
**Co-medication**		
Diuretics	0.96 (0.91–1.01)	0.107
Beta-blockers	1.06 (1.00–1.12)	0.035
Calcium channel blockers	1.08 (1.02–1.15)	0.008
RAAS inhibitors	1.06 (1.01–1.12)	0.028
Lipid-lowering agents	1.05 (1.00–1.12)	0.073

Subsequent prescriptions comprise all anticoagulant prescriptions following each patient's first anticoagulant prescription among 87,803 AF patients with at least one anticoagulant prescription. Covariates were determined at the date of the patient's first anticoagulant prescription. Logistic regression with generalized estimating equations (GEE; independent working correlation, robust standard errors, clustering by patient). COPD: chronic obstructive pulmonary disease; TIA: transient ischemic attack; RAAS: renin-angiotensin-aldosterone system; OR: odds ratio; CI: confidence interval.

Table S6. Switching between anticoagulant classes among 87,803 AF patients.

Characteristic	n (%)
Patients with a switch to another class	6456 (7.4)
Patients with three different classes	134 (0.2)
**Direction of the first switch (% of patients with a switch)**	
Vitamin K antagonists → direct factor Xa inhibitors	4229 (65.5)
Direct factor Xa inhibitors → vitamin K antagonists	850 (13.2)

Direct thrombin inhibitors → direct factor Xa inhibitors	773 (12.0)
Direct factor Xa inhibitors → direct thrombin inhibitors	450 (7.0)
Vitamin K antagonists → direct thrombin inhibitors	100 (1.5)
Direct thrombin inhibitors → vitamin K antagonists	54 (0.8)
**Timing and dispensing**	
Days from the first prescription to the switch, median (IQR)	671 (222–1425)
Last prescription of the previous class not dispensed within 30 days	738 (11.5)
First prescription not dispensed: patients with a switch	796 (12.3)
First prescription not dispensed: patients without a switch	6448 (7.9)

The last prescription of the previous class before the switch could be identified for 6437 patients. IQR: interquartile range.
